# The Fecal Viral Flora of Wild Rodents

**DOI:** 10.1371/journal.ppat.1002218

**Published:** 2011-09-01

**Authors:** Tung G. Phan, Beatrix Kapusinszky, Chunlin Wang, Robert K. Rose, Howard L. Lipton, Eric L. Delwart

**Affiliations:** 1 Blood Systems Research Institute, San Francisco, California, United States of America; 2 Department of Laboratory Medicine, University of California at San Francisco, San Francisco, California, United States of America; 3 Department of Viral Diagnostics, National Center for Epidemiology, Budapest, Hungary; 4 Division of Infectious Diseases, Stanford University Medical Center, Stanford, California, United States of America; 5 Department of Biological Sciences, Old Dominion University, Norfolk, Virginia, United States of America; 6 Department of Neurology and Microbiology-Immunology, University of Illinois at Chicago, Chicago, Illinois, United States of America; Columbia University, United States of America

## Abstract

The frequent interactions of rodents with humans make them a common source of zoonotic infections. To obtain an initial unbiased measure of the viral diversity in the enteric tract of wild rodents we sequenced partially purified, randomly amplified viral RNA and DNA in the feces of 105 wild rodents (mouse, vole, and rat) collected in California and Virginia. We identified in decreasing frequency sequences related to the mammalian viruses families *Circoviridae*, *Picobirnaviridae*, *Picornaviridae*, *Astroviridae*, *Parvoviridae, Papillomaviridae*, *Adenoviridae*, and *Coronaviridae*. Seventeen small circular DNA genomes containing one or two replicase genes distantly related to the *Circoviridae* representing several potentially new viral families were characterized. In the *Picornaviridae* family two new candidate genera as well as a close genetic relative of the human pathogen Aichi virus were characterized. Fragments of the first mouse sapelovirus and picobirnaviruses were identified and the first murine astrovirus genome was characterized. A mouse papillomavirus genome and fragments of a novel adenovirus and adenovirus-associated virus were also sequenced. The next largest fraction of the rodent fecal virome was related to insect viruses of the *Densoviridae*, *Iridoviridae*, *Polydnaviridae*, *Dicistroviriade*, *Bromoviridae*, and *Virgaviridae* families followed by plant virus-related sequences in the *Nanoviridae*, *Geminiviridae*, *Phycodnaviridae*, *Secoviridae*, *Partitiviridae*, *Tymoviridae*, *Alphaflexiviridae*, and *Tombusviridae* families reflecting the largely insect and plant rodent diet. Phylogenetic analyses of full and partial viral genomes therefore revealed many previously unreported viral species, genera, and families. The close genetic similarities noted between some rodent and human viruses might reflect past zoonoses. This study increases our understanding of the viral diversity in wild rodents and highlights the large number of still uncharacterized viruses in mammals.

## Author Summary

Rodents are the natural reservoir of numerous zoonotic viruses causing serious diseases in humans. We used an unbiased metagenomic approach to characterize the viral diversity in rodent feces. In addition to diet-derived insect and plant viruses mammalian viral sequences were abundant and diverse. Most notably, multiple new circular viral DNA families, two new picornaviridae genera, and the first murine astrovirus and picobirnaviruses were characterized. A mouse kobuvirus was a close relative to the Aichi virus human pathogen. This study significantly increases the known genetic diversity of eukaryotic viruses in rodents and provides an initial description of their enteric viromes.

## Introduction

The order *Rodentia* is the single largest group of mammalian species accounting for 40% of all mammal species [1]. There are ca 2200 living rodent species, including mice, rats, voles, squirrels, prairie dogs, beavers, chipmunks, and guinea pigs. Many rodents have mixed diets but some eat mostly seeds or green vegetation. Rodents are known to vector more than 60 known human infectious diseases [2]. Some rodents live in close association with humans offering numerous opportunities for cross-species viral transmission through their urine, feces, or their arthropod ectoparasites such as ticks, mites, and fleas [2]–[8].

Rodents have been associated with numerous viruses including members of the *Arenaviridae*, *Reoviridae*, *Togaviridae*, *Picornaviriade*, and *Flaviviridae* families [2], [9]–[12]. The hantavirus pulmonary syndrome (HPS), an infection with an exceptionally high mortality first identified in the southwestern United States, was recognized as a zoonotic viral infection with Sin Nombre virus (SNV) in the Hantavirus genus in the *Bunyaviridae* family originating from deer mouse (*Peromyscus maniculatus*) [13]. Since then, HPS has been identified throughout the United States [14]–[17] with SNV responsible for most cases [18]–[23]. Deer mice captured in Montana showed an SNV antibody prevalence of approximately 11% [24]. Other members of the *Hantavirus* genus transmitted from rodents include Hantaan, Dobrava-Belgrade, Seoul, and Puumala viruses, causing hemorrahagic fever with renal syndrome (HFRS) worldwide [25]–[31]. HFRS was endemic in 28 of 31 provinces of China and is considered a major public health concern. Over 1,200 HFRS cases occurred in 2007 in China [32], [33]. It was reported that there were approximately 8,300 patients with HFRS in Inner Mongolia and 261 (3.14%) died during 1955–2006 [33]. Recently, Seoul virus was detected in 47 of 649 Norway rats (*Rattus norvegicus*) [34]. Several vole species (*Microtus arvalis*, *Pitymys subterraneus*, and *M. subterraneus*) have been linked with Tula virus also in the *Hantavirus* genus [35]–[39]. In Switzerland, acute infection with Tula virus was found in a 12-year-old boy after a rodent's bite [40]. Tick-borne encephalitis virus (TBEV) in the *Flavivirus* genus of the family *Flaviviridae* can cause fatal encephalitis in humans [41]–[43]. Several rodent species such as voles (*Microtus agrestis* and *Myodes glareolus*), field mice (*Apodemus agrarius*) are natural hosts of ticks that cause TBE [43], [44]. Lassa fever, an acute viral hemorrhagic fever first described in 1969 in Nigeria, is caused by Lassa virus, a member of the family *Arenaviridae* [45]. Its primary animal host is the Multimammate mouse (*Mastomys [Praomys] natalensis*) [3]. Lassa fever, endemic in West Africa, causes 30,000–500,000 cases and 5,000 deaths annually [46].

Because of their health and economic impact there is a growing awareness of emerging (and re-emerging) zoonotic infections [2], [7]. Increased interactions between rodents and humans occur when people build homes in wildlife habitat or conduct more recreational activities there [2], [47], [48]. Irruptions in density of rodent hosts, as occurred prior to the SNV infections in the southwestern US, also increase the risk of human viral exposure [49]. Viral surveys in wild and domesticated animals with extensive contacts with humans can be used to monitor for the presence of known zoonotic viruses or closely related viral species and to provide a baseline of the viruses present to help detect future changes associated with disease outbreaks. The identification of animal viruses closely related to human viruses also provides information regarding past successful zoonoses. To assist in these goals we performed an initial characterization of the fecal viromes of rodents from two locations in the US.

## Results

### Viral metagenomic overview

**Figure 1:**
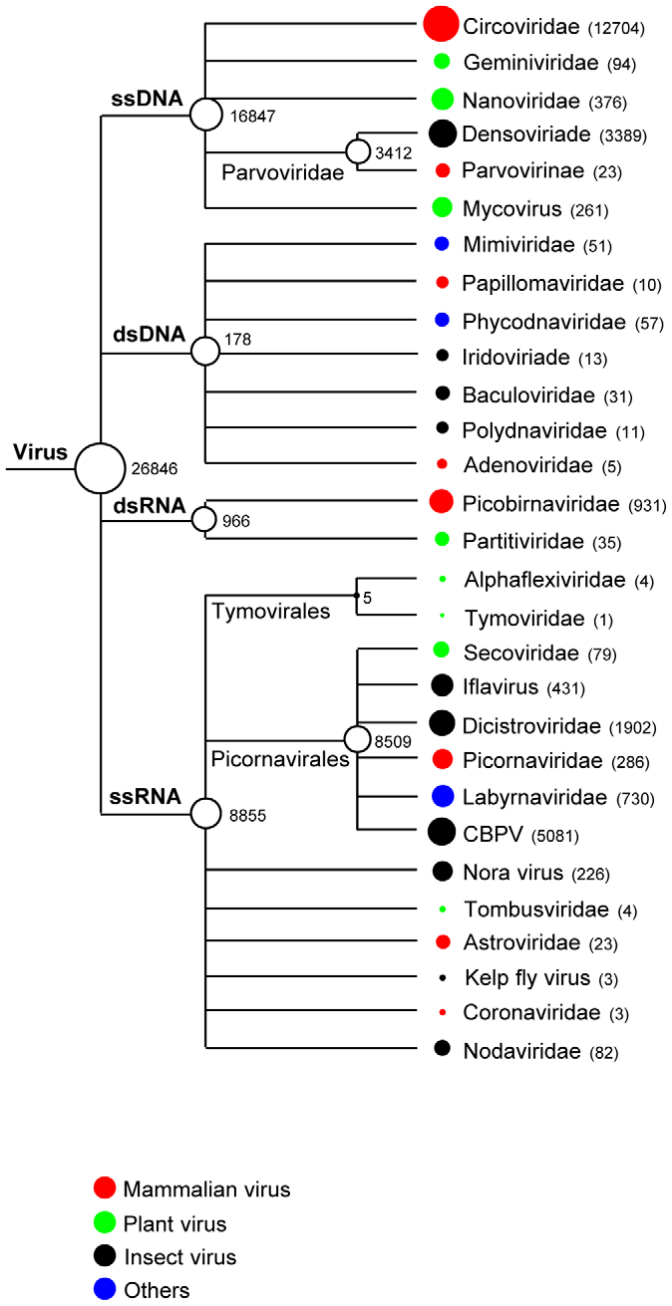
Taxonomic classification of sequences with similarity to eukaryotic viruses. Circles located next to taxa are logarithmically proportional to the total number of sequence reads with BLASTx E<10^−5^. The numbers of sequence read are also included.

Viral particles in fecal samples were purified by filtration, digested with DNase and RNase enzymes to remove unprotected nucleic acids, amplified by random RT-PCR, and subjected to 454 pyrosequencing. A total of 1,441,930 sequence reads with an average of 177-bp were generated from the extracted nucleic acids in the present study and the sequences from each animal were assembled de novo into contigs of variable length. Both singlets and contigs longer than 100-bp were then classified using BLASTx and BLASTn as likely virus, phage, bacteria, or eukaryota based on the taxonomic origin in the annotation of the best-hit sequence (E score <10^−5^) with the GenBank non-redundant database. Fecal samples of mice, voles, and a woodrat revealed a large degree of microbial diversity. 26,846 sequence reads had best matches with viral protein, RNA or DNA sequences as shown in Figure 1. There were also ∼585,000 sequences for bacteria, 30,400 for eukaryota, and 154,000 for phage. A large proportion of the total reads (45%) did not have any significant hits to nucleotide or amino acid sequences in GenBank in agreement with viral metagenomic studies of feces from bats, turkeys, and humans [50]–[52].

### Mammalian viruses

The largest proportion of the rodent fecal virus-related sequences (52%) was related to mammalian viruses, with 91% of these being related to DNA viruses. Viral sequences related to single-stranded DNA viruses in the *Circoviridae* were abundant, comprising 90% of the mammalian DNA virus-like sequence reads. DNA viruses in the *Parvovirinae subfamily*, *Papillomaviridae*, and *Adenoviridae* families were also detected. RNA viral sequences were mostly related to the families *Picobirnaviridae* and *Picornaviridae*. A few RNA virus sequences (n = 23) related to the family *Astroviridae* were also identified. While some sequences showed >90% similarity at the amino acid level with known viruses, the majority exhibited <70% similarity. We further characterized some of these novel mammalian virus-like sequences by full or near full genome sequencing and compared them to their closest relatives by phylogenetic analyses. Partial viral genomes were similarly analyzed.

### Mouse papillomavirus

Papillomaviruses (PVs) are a highly diverse family of double-stranded circular DNA genomes ca 8-kb in size. PVs are known to infect a wide variety of mammals, as well as birds and reptiles. Some PV types cause benign or malignant epithelial tumors of the skin and mucous membranes in their natural hosts, while others are commonly present in the healthy skin of healthy humans, as well as a range of different animal species. PVs are highly species-specific and rarely transmitted between species. More than 100 human PV types have been detected, and the genomes of more than 80 have been completely sequenced [53], [54]. Only a few full genomes of PVs have been reported in non-human species.

**Figure 2:**
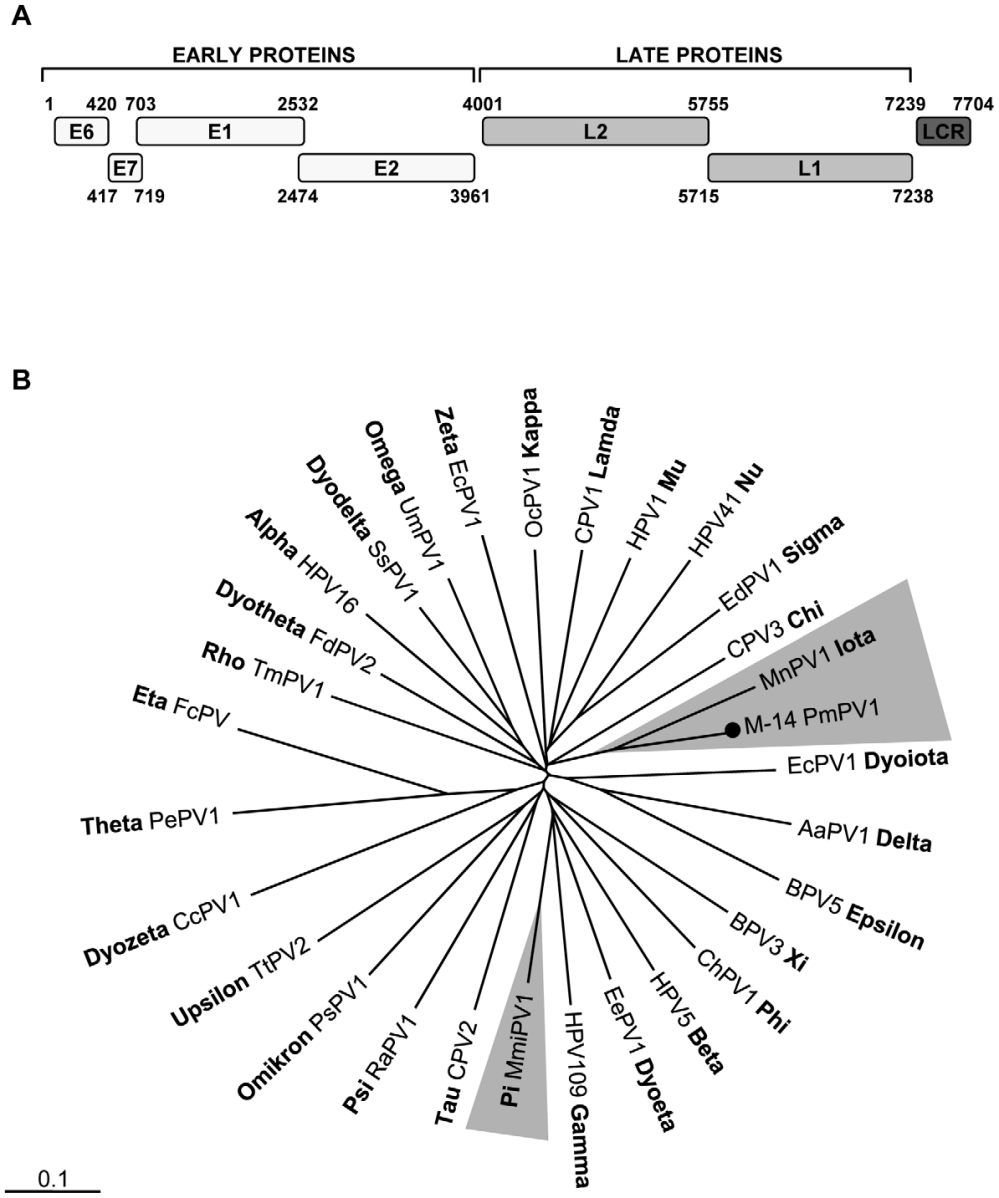
Mouse papillomavirus. **A**. Genome organization of a novel mouse papillomavirus. **B**. Phylogenetic tree generated with concatenated L1 and L2 capsid proteins of PmPV1_M-14 and representatives of all genera in the family *Papillomaviridae*. The novel mouse papillomavirus in this study is labeled with a black circle. The other murine papillomavirus MmiPV1 is highlighted in grey. The scale in this and every tree indicates amino acid substitutions per position.

We characterized the full-length genome of the deer mouse (*Peromyscus maniculatus*) PV type 1, hereafter referred to as PmPV1 (GenBank JF755418). The complete circular PmPV1 genome was 7,704-bp, with a GC content of 51%. Six distinct ORFs on the same coding strand were identified, including the early genes E6, E7, E1, and E2 and the late genes L2, and L1 (Figure 2A). Analysis of the deduced amino acid sequences revealed two characteristic zinc-binding domains (C-X_2_-C-X_29_-C-X_2_-C) in E6, separated by 36 amino acids. E7 also contained a zinc-binding domain and the conserved retinoblastoma tumor suppressor-binding motif (L-X-C-X-E). The C-terminal region of E1 protein had an ATP-dependent helicase motif (GPPDTGKS) and also contained a cyclin interaction RXL motif required for viral replication. The long control region (LCR) between the end of the L1 gene and the start of the E6 gene, was 469 bp. In the C-terminus of LCR, two consensus E2-binding sites (ACC-X6-GGT) were present. The TATA box (TATAAA) of the E6 promoter was located at position 7661 and a polyadenylation site (AATAAA; nt 7240) for processing of the L1 and L2 capsid mRNA transcripts were found at the N-terminus of the LCR. Taken together, many of the classic PV specific elements were identified in PmPV1.

The family *Papillomaviridae* currently contains at least 29 genera and mouse PVs have been found in the *Pipapillomavirus* and *Iotapapillomavirus genera* [53]. Phylogenetic analysis of the complete L1 protein was performed. PmPV1 shared the same root as the Multimammate mouse (*Mastomys natalensis*) PV type 1 (MnPV1) in the *Iotapapillomavirus* genus (Figure 2B). PmPV1 had the highest L1 similarity of 67% to the L1 of MnPV1 (Table S1). In addition, the closest amino acid similarities of other major PmPV1 proteins (E1, E2 and L2) were also to MnPV1. According to the International Committee on Taxonomy of Viruses (ICTV), different PV species share between 60% and 70% of nucleotide sequence similarity in the L1 ORF [53]. PmPV1 is therefore a proposed new PV species within the *Iotapapillomavirus* genus sharing 63% similarity with MnPV1.

### Rodent circo-like viruses

Circular ssDNA viruses known to infect animals have the smallest viral genomes and are classified in the *Circoviridae* family and the unassigned genus *Anellovirus*. Circular ssDNA genome infecting plants belong to the *Germiniviridae* and *Nanoviridae* families. Despite very distinct host-specificities, these viruses share conserved motifs in their Replication initiator proteins (Rep) including a helicase domain [55]. Several circovirus-like genomes were also recently characterized from reclaimed water and marine environments [56], [57] and directly from a single cell of a marine protist [58]. Rep-like sequences were found in feces from 23% (12/52) of mice, 63% of voles (33/52), and 100% of cotton rat (1/1). Seventeen full circular DNA viral genomes were then sequenced using inverse PCR targeting the initial Rep sequence matches. The GenBank accession numbers are in Table S2. One genome was derived from feces of the Woodrat *Neotoma cinerea*, four from the mice *Peromyscus maniculatus* and *Mus musculus* and twelve from the voles *Microtus pennsylvanicus*. The smallest genome was 1,124-bp and the largest 3,781-bp.

**Figure 3:**
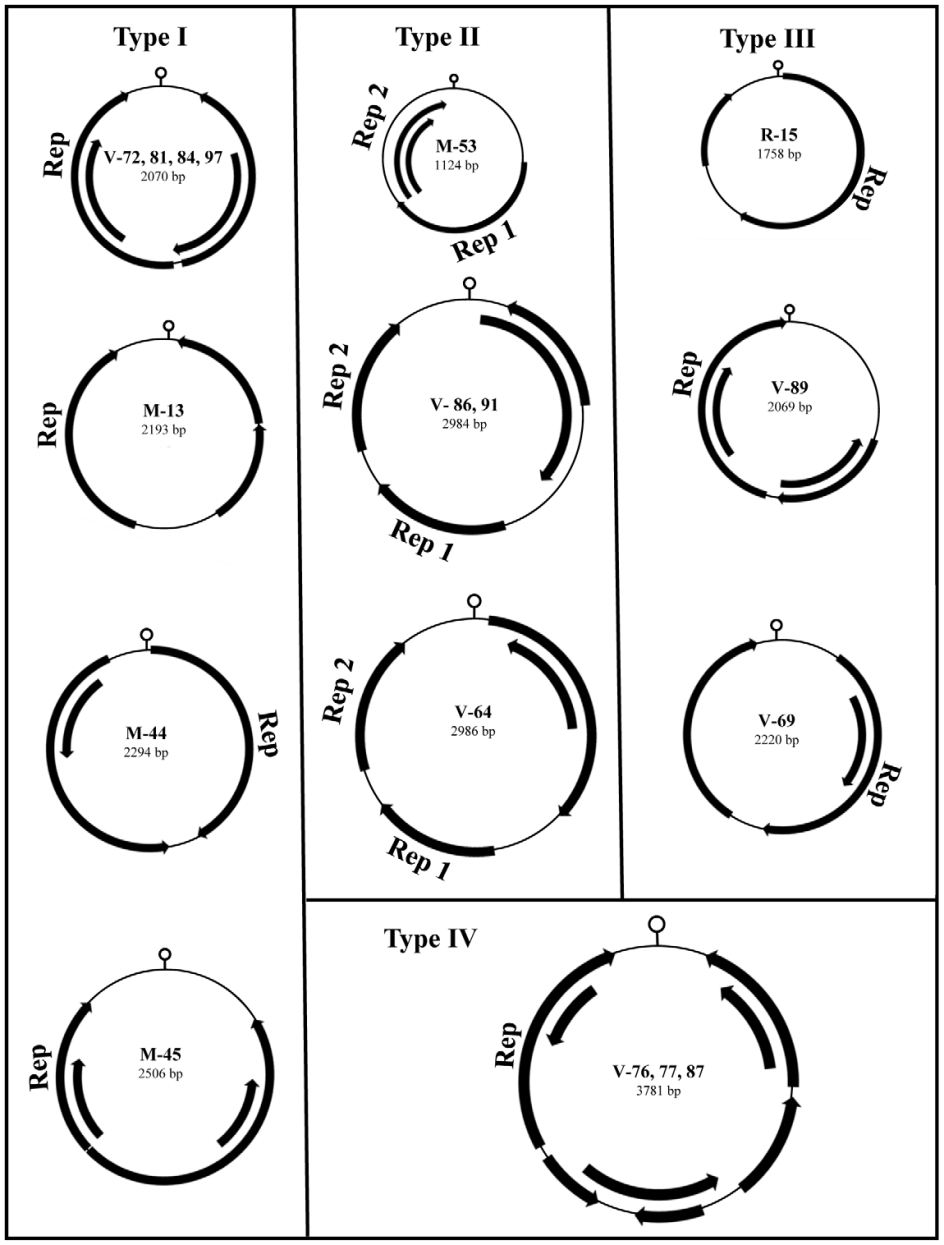
Genomic organization of RodSCVs. The novel rodent stool-associated circular viruses (RodSCVs) are classified into four different genomic organization categories. The location of putative Rep genes and other ORFs (greater than 100 amino acids long) are indicated by arrows.

These replicase-containing circular genomes could be placed into four different types of ORF organization following the classification of Rosario et al [56] (Figure 3). The type I genome had features similar to circoviruses and was characterized by a small circular DNA genome (approximately 2-kb), with the Rep protein and an unknown major ORF in opposite orientation. However, a majority of the type I genome had a stem-loop structure located in the 3′ downstream intergenic region distinct from the 5′ intergenic location seen in circoviruses, nanoviruses, and geminiviruses. The type II genome had the unique feature of encoding two separate Rep ORFs. The type III genomes contained two major ORFs in the same orientation in a manner similar to anelloviruses, a highly diverse but phylogenetically unrelated group of viruses with circular DNA genomes. The type IV genome had the largest genomes of nearly 4-kb, with up to eight ORFs. Due to their diverse genomic architectures, we preliminarily named these circular DNA genomes rodent stool-associated circular viruses (RodSCVs).

A DNA stem-loop containing a conserved nonamer is thought to have an important role in initiating rolling-cycle and a binding site for the Rep protein during circovirus replication [59]–[61]. The nonamer sequences in RodSCVs were slightly different from each other, but shared 5–7 nucleotide identity with those in circoviruses, nanoviruses, and geminiviruses [62]. Interestingly, RodSCV-M-13, M-45, and V-89 had the same nonamer sequence GGGTAATAC although they differed in genomic size and organization (Table S2).

The Rep proteins in RodSCVs possessed conserved motifs (DRYP and WWDGY) but not the DDFYGW motif typical of circoviruses. The Rep proteins in RodSCVs contained at least one well-known superfamily domain, either viral Rep, RNA helicase, or Gemini-AL1. In the type I genome, M-45 carried a homologue of the viral Rep superfamily; however, M-13, -44, V-72, -81, -84 and -97 contained both the viral Rep plus the RNA helicase superfamily domains within a single Rep ORF. By BLASTx, the Rep proteins of M-44 and 45 showed the most significant hits (E-value  = 1×10^−73^ and 2×10^−19^, respectively) with circovirus-like genomes found in the environment [56] (Table S2). The Rep proteins of RodSCV-M-13, -44, V-72, -81, -84 and -97 showed the top BLAST hits (E-value  = 4×10^−35^ and 2×10^−82^, respectively sharing 20–52% protein similarity) to the Rep protein of *Giardia intestinalis*, a major diarrhea causing parasite in humans. In the type II genome, M-53 had both the viral Rep and the RNA helicase superfamilies in two separate Rep ORFs. V-64, -86 and -91 did not contain any recognizable viral Rep superfamily protein domain, but their Rep-like proteins showed best BLASTx matches with the Rep of the plant geminiviruses. In the type III and IV genomes, RodSCV-R-15, V-76, -77 and -87 contained the Gemini-AL1 superfamily motif that is commonly found in geminiviruses (Table S2). None of the other ORFs in the all RodSCVs showed detectable similarities to known protein superfamilies or significant matches in GenBank.

**Figure 4:**
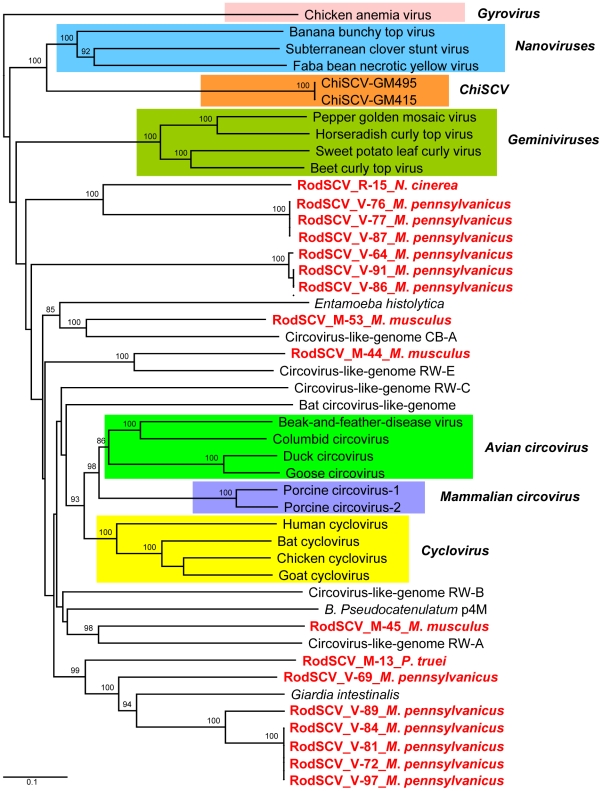
Phylogenetic analysis of Rep proteins of RodSCV and single stranded circular viral genomes. Phylogenetic tree obtained from amino acid sequences of complete Rep genes of members of the families *Geminiviridae, Nanoviridae, Circoviridae*, *Gyrovirus*, *Canarypox virus*, *Circovirus-like genomes*, *Bifidobacterium pseudocatenulatum* plasmid p4M, *Giardia intestinalis*, and *Entamoeba histolytica*. The novel RodSCVs in this study are highlighted in red.

In order to phylogenetically classify the RodSCVs their Rep proteins were aligned to those of circoviruses, cycloviruses, geminiviruses, nanoviruses, gyrovirus (Chicken anemia virus), environmental circovirus-like genomes, *Bifidobacterium pseudocatenulatum* plasmid p4M, and the protozoans *Giardia intestinalis* and *Entamoeba histolytica*. The RodSCV Rep were located on separate branches with some clustering with circovirus-like genomes derived from marine and reclaimed waters while other clustered with an integrated Rep in the genome of the parasitic protozoan *Giardia intestinalis* (Figure 4). Theses findings indicated that the RodSCVs likely represent several novel viral families.

### Mouse kobuvirus

The Aichi picornavirus was first identified in human cases of oyster-associated gastroenteritis in 1989 [63]–[65]. Aichi virus, a second species of human kobuvirus named salivirus/klassevirus also associated with diarrhea [66]–[70], bovine kobuvirus, porcine kobuvirus and a partial genome from a bat constitute the *Kobuvirus* genus in the family *Picornaviridae (* http://www.picornaviridae.com/kobuvirus/kobuvirus.htm *)*. The reference Aichi virus genome is approximately 8-kb and similar to other picornaviruses contains a single large ORF that encodes a large polyprotein of 2,433 amino acids proteolytically cleaved into structural and non-structural proteins.

**Figure 5:**
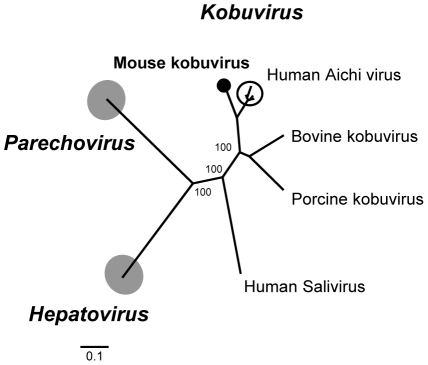
Phylogenetic analysis of mouse kobuvirus. Phylogenetic tree generated with complete 3D^pol^ protein of members of the genera *Kobuvirus, Hepatovirus and Parechovirus* in the family *Picornaviridae*. The human Aichi viruses are encircled. The novel mouse kobuvirus in this study is labeled by a black circle.

We found several sequence reads from two mouse (*Peromyscus crinitus* and *P. maniculatus*) fecal samples with similarities to Aichi virus. The sequence reads shared a high nucleotide similarity of 93% to each other, suggesting that the picornaviruses in these mice were from the same species. We successfully acquired the complete genome (8,171-bp, excluding the poly(A) tail) of the kobuvirus M-5 from *Peromyscus crinitus* mouse (GenBank JF755427), including 5′ UTR (610-bp), polyprotein ORF (2,439-aa) and 3′ UTR (244-bp). An optimal Kozak sequence, RNNAUGG (ATCATGG), was found at the beginning of the translated polyprotein. The leader (L) protein, located in the amino-terminal part of the polyprotein, showed the closest match (68%) to the L of human Aichi virus. The N-terminal zinc finger protein-binding motif C-X-H-X(6)-C-X(2)-C essential for the cytosol-dependent phosphorylation cascade found in L proteins of the *Cardiovirus* genus, such as Encephalomyocarditis virus and Theiler's murine encephalomyelitis virus could not be found in the mouse kobuvirus [71], [72]. In the 2C protein a highly conserved nucleotide-binding domain of the helicase (GPPGTGKS) was identified. In addition, the RNA-binding domain GLCG and the H-D-C catalytic triad were present at amino acid positions 42, 84 and 143 in the 3C^pro^ region as were the characteristic RdRp KDELR, YGDD and FLKR motifs in 3D^pol^. Phylogenetic analysis of 3D^pol^ showed that the mouse kobuvirus was the closest relative to the human Aichi virus (Figure 5). This finding was also supported by pair-wise comparison in which mouse Kobuvirus M-5 had 81–84% similarities to human Aichi virus and 53–63% similarities to bovine kobuvirus at the amino acid level for the P1, P2 and P3 regions (Table S3). Genome analysis therefore indicated that the M-5 should be considered a new species and the first murine kobuvirus.

Molecular epidemiology of human Aichi virus worldwide has been performed using specific primers targeting the 3CD junction [73]–[75]. In order to understand the prevalence of Aichi-like viruses in rodents, we designed consensus primers based on 2C-3B regions conserved between rodent and human Aichi viruses and used RT-nPCR to screen other rodent fecal samples. Two additional mice *(Peromyscus crinitus* and *P. maniculatus*) were positive for Aichi virus. Their nucleotide sequences over that region (∼700-bp) were 94–95% similar to the mouse kobuvirus with which they also clustered phylogenetically (data not shown).

### Mouse Sapelovirus

We detected a Sapelovirus-related sequence encoding 207 amino acids in house mouse (*Mus musculus*) feces M-58 (GenBank JF755421). This sequence covered about 25% of the capsid encoding P1 region and showed the best amino acid similarity (53%) to a porcine Sapelovirus. According to the ICTV, members of a picornavirus genus should share at least 40% amino acid similarity in the P1 region. Phylogenetically this sequence was also related to the Sapelovirus genus, indicating that it represents a fraction of the first reported murine Sapelovirus genome (Figure S1).

### Proposed new mouse picornavirus genus

**Figure 6:**
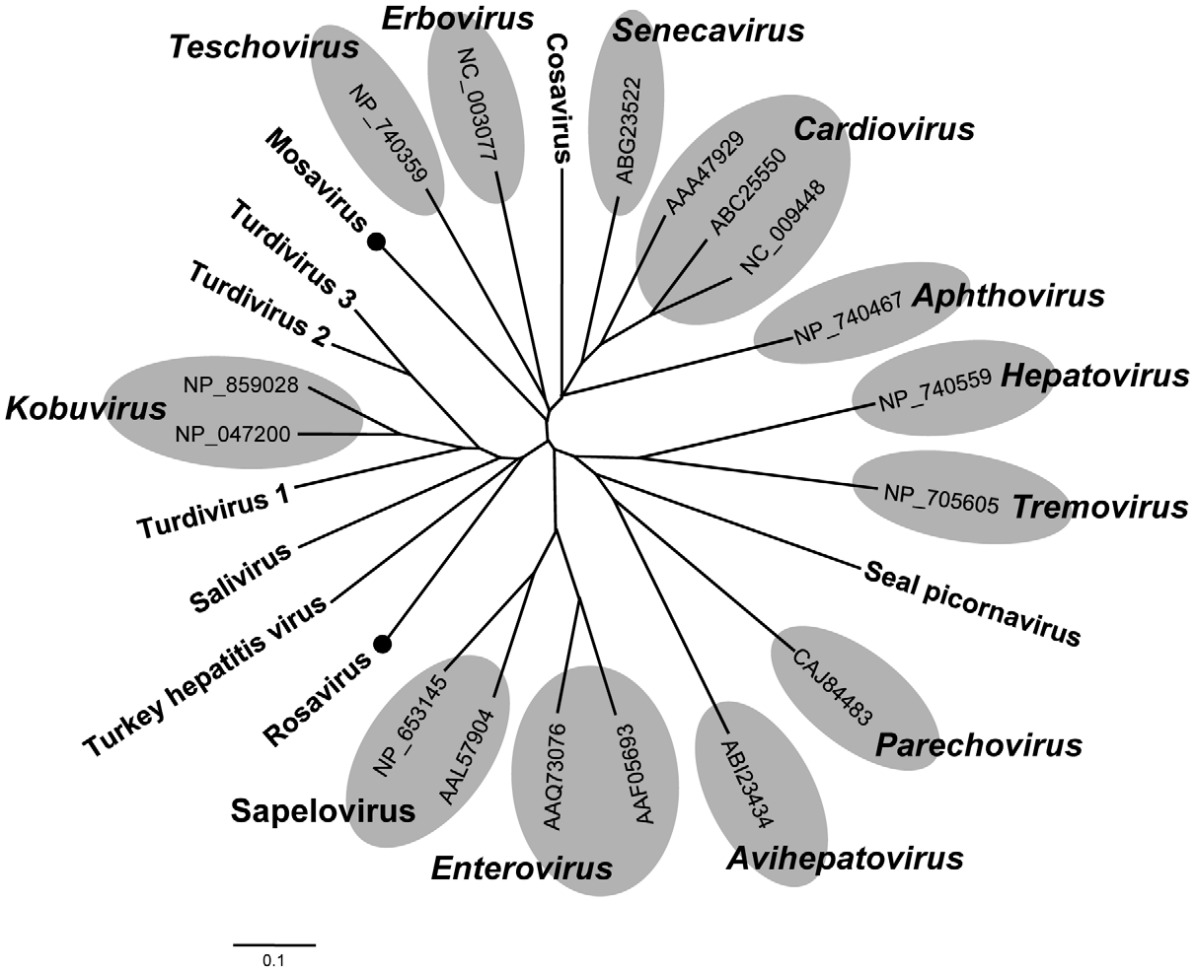
Phylogenetic analysis of Mosavirus and Rosavirus. Phylogenetic tree obtained from amino acid sequences of complete 3D proteins of all taxonomic genera in the family *Picornaviridae*. Mosavirus and Rosavirus are labeled with black circles.

The family *Picornaviridae* currently consists of 12 genera, although recently characterized genomes are expected to nearly double that number (www.picornaviridae.com). In this study, we found a novel picornavirus in the feces of a canyon mouse (*Peromyscus crinitus*) we temporarily named Mosavirus for mouse stool associated picornavirus. We successfully acquired the nearly complete genome (6934-bp, excluding the poly(A) tail) of Mosavirus (GenBank JF973687) including a partial 5′ UTR (138-bp), complete polyprotein ORF (2235-aa) and complete 3′ UTR (88-bp). The hypothetical cleavage map of the Mosavirus polyprotein was derived from alignments with other known picornaviruses. Similar to Cardioviruses and Senecavirus, Mosavirus had a conserved cleaved site Q↓GN for a putative L protein preceding the capsid region. The P1 polypeptide (758-aa) contained the rhv-like superfamily and best BLASTx match to Theiler's encephalomyelitis virus in *Cardiovirus* genus, sharing 37% aa similarity. The P1 was hypothesized to be cleaved at VP4/VP2 (M↓D), VP2/VP3 (Q↓G) and VP3/VP1 (Q↓G). The VP4 was found to have the GXXX[T/S] myristylation site. The P2 polypeptide (501-aa) encoded non-structural proteins cleaved at 2A/2B (G↓P) and 2B/2C (Q↓G). BLAST showed that the P2 region had the highest aa similarity of 36% to the Saffold virus belonging to *Cardiovirus* genus. Similar to cardioviruses the 2A protein in Mosavirus had the conserved NPGP motif for 2A-mediated cleavage at the 2A/2B junction. The 2A protein was predicted to be 36 amino acids shorter than those of any known cardioviruses (∼150-aa). The 2C protein contained some characteristic features of picornaviruses such as the RNA-helicase superfamily, NTPase and helicase motifs. The P3 polypetide encoded proteins 3A, 3B (VPg, small genome-linked protein), 3C^pro^ (protease) and 3D^pol^ (RNA-dependent RNA polymerase). P3 with 822 amino acids in length was cleaved at 3A/3B (E↓G), 3B/3C (Q↓G) and 3C/3D (Q↓G). 3C^pro^ contained the peptidase C3 superfamily and conserved GXCG motifs. Some conserved YGDD, FLKR and GG[LMN]PSG motifs were identified in the 3D protein. The pair-wise amino acid sequence analysis demonstrated that the P3 polyprotein in Mosavirus shared very low aa similarity, less than 29%, to the genetically-closest picornaviruses (Table S4). Based on the 3D^pol^ phylogenetic tree (Figure 6) and genetic distance calculations, Mosavirus is not significantly linked to any recognized or proposed genera. According to the ICTV, the member of a picornavirus genus should share >40%, >40% and >50% amino acid similarity in P1, P2 and P3 regions respectively. The similarities over these three regions in Mosavirus were less than 40% at the amino acid level to those of any reported piconaviruses. Mosavirus is therefore proposed as a novel genus in the family *Picornaviridae*.

### A second proposed new picornavirus genus

In the same house mouse feces where Mosavirus was found, we also found another picornavirus temporarily named Rosavirus for rodent stool associated picornavirus. A genome fragment of 3,956-bp was sequenced, including a partial 2C gene, the complete P3 region and a complete 3′ UTR (GenBank JF973686). The 2C segment contained the conserved NTPase motif GXXGXGKS (GGPGCGKS) and helicase motif DDLGQ typical of picornaviruses. The P3 region of Rosavirus was hypothesized to be cleaved at 3A/3B (E↓G), 3B/3C (Q↓I) and 3C/3D (Q↓G). The 3A (106-aa) and 3B (31-aa) proteins had typical lengths but did not show any detectable sequence similarity to other picornaviruses. 3B did have a conserved tyrosine at position 3 and another conserved glycine at position 5, consistent with its function as the genome-linked protein, VPg.

The 3C, with 206 amino acids, had the H-D-C catalytic triad at positions 47, 90, and 158, followed by 15 amino acids downstream of the GIH motif, similar to the substrate-binding site of chymotrypsin-like proteases. By BLAST, 3C contained the peptidase C3 superfamily and was genetically closest to turkey hepatitis virus, sharing 30% amino acid similarity. Rosavirus 3D contained conserved RdRp motifs KDELR, YGDD and FLKR. Interestingly, the 3D protein in Rosavirus has a mutated motif GAMPSG compared with conserved GG[LMN]PSG in other picornaviruses. The 3D protein in Rosavirus was most closely related to the 3D of avian turdivirus 2, sharing 44% amino acid similarity. In addition, Rosavirus had the longest reported 3′ UTR of 795-bp in length.

The P3 region in Rosavirus showed very low amino acid similarity (<31%) to the closest picornavirus (Table S4). The P3 regions of Rosavirus and Mosavirus shared only 23% similarity at the amino acid level. The 3D^pol^-based phylogenetic grouping demonstrated that Rosavirus shared a monophyletic root with members of the genus *Kobuvirus*, turdiviruses and turkey hepatitis virus (Figure 6). A similar topology was found in a 3C^pro^-based phylogenetic analysis (data not shown). Based on genetic distance criteria Rosavirus is therefore a candidate prototype for a novel genus in the family *Picornaviridae*.

### Rodent picobirnaviruses

Picobirnavirus (PBV), the only genus in the new *Picobirnaviridae* viral family, has a bi-segmented dsRNA genome. The large RNA segment encodes the capsid protein while the small segment encodes the viral RdRp. Originally found in the intestines of rat [76] PBV has since been found in numerous mammals [77]. In humans, ca 20% of fecal diarrheal samples in the Netherlands were positive for PBV [78]. This virus has also been reported as a causative agent of gastroenteritis in HIV-positive patients [79], [80]. Based on the small segment sequence, PBV has been classified into two genogroups, I and II [81]. However, there are few full-length sequences of this segment.

**Figure 7:**
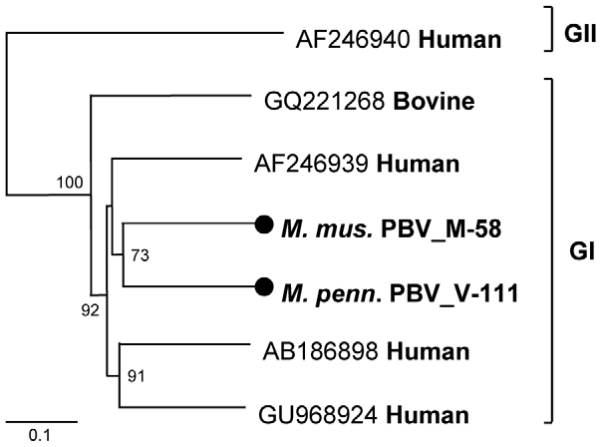
Phylogenetic analysis of rodent picobirnaviruses. Phylogenetic tree obtained from nearly full-length RdRp protein of genogroup I and II picobirnaviruses in the family *Picobirnaviridae*. The novel picobirnaviruses are labeled by black circles.

23% of feces from mice (12/52) and 19% from voles (10/52) contained PBV related sequences, the second highest prevalence and number of mammalian virus-like sequences after the Circoviridae-like reads (Figure 1). Previous reports indicated that the genogroup I was predominant in humans [78], [82]. Two fecal specimens had large number of PBV-like reads that assembled into nearly full-length RdRp for two strains, M-58 (house mouse *Mus musculus*) (412-aa at GenBank JF755419) and V-111 (vole *Microtus pennsylvanicus*) (414-aa at GenBank JF755420). Amino acid sequence alignment of nearly full-length RdRp allowed the construction of a phylogenetic tree using all other available sequences of similar length (Figure 7). M-58 and V-111 clustered with strains in genogroup I. M58 and V-111 were 63% similar at the amino acid level to each other and only 54% to 63% similar to human and bovine PBVs in genogroup I. These two picobirnaviruses therefore represent new PBV species, the first reported in mice and voles.

### Mouse astrovirus

The family *Astroviridae* consists of two genera, namely, *Mamastrovirus* and *Avastrovirus*, that infect mammalian and avian species, respectively. Astrovirus (AstV), a member of the *Mamastrovirus* genus, is a small, non-enveloped, single-strand RNA virus that has been associated with human gastroenteritis and detected in association with other enteric pathogens [83]. The genome of astroviruses range from 6.1 to 7.3-kb and contains ORF1a, 1b, and 2, coding for serine protease, RdRp, and capsid protein, respectively. Astroviruses have been reported in fecal specimens from humans, bats, rats, pigs, cattle, and other animals [51], [84]-[88]. Astroviruses recently detected in human specimens were genetically related to animal astroviruses, indicating their possible zoonotic origins [89]–[91].

**Figure 8:**
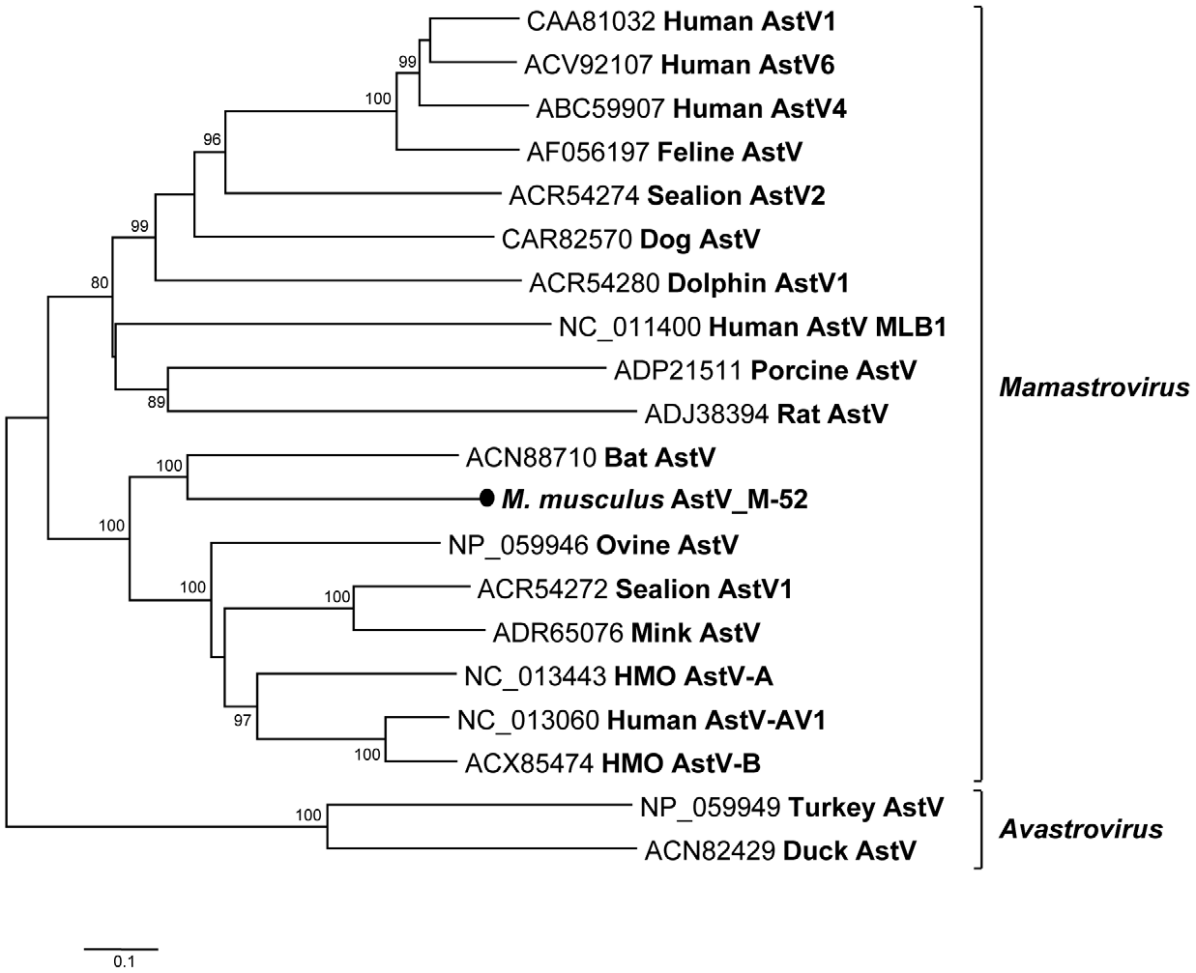
Phylogenetic analysis of mouse astrovirus. Phylogenetic tree obtained from complete ORF2 protein of astroviruses in the family *Astroviridae*. The novel astrovirus is labeled with a black circle.

We sequenced the complete genome of an astrovirus from house mouse (*Mus musculus*) feces (M-52) (GenBank JF755422). The 6,519-bp genome length included a 14 bases 5′ UTR, ORF1a (848-aa), ORF1b (545-aa), ORF2 (707-aa) and 3′ UTR (332-nt, excluding the poly(A) tail). ORF1a contained a trypsin-like serine protease domain and ORF1b encoded RdRp following a -1 ribosomal frameshift induced by the presence of a heptameric “slippery sequence” AAAAAAC. The consensus promoter initiating ORF2 subgenomic RNA synthesis in the mouse astrovirus was identified as CUUUGGAGGGGUGGACCAAGAGGAGACAAUGGC (start codon in boldface). The 3′ UTR (332-nt) was longer than that of previously described astroviruses. This region contained the highly conserved 35-nt motif also found in other astroviruses from human, bat, porcine, and duck, in the avian picornavirus turdivirus as well as in a dog norovirus [92], [93]. Phylogenetic analysis based on the capsid protein showed that M-52 was linked to an astrovirus clade including bat, human, mink, and sheep astroviruses (Figure 8). BLASTx searches demonstrated that complete ORF1b and ORF2 of M-52 were most closely related to those of a bat astrovirus, sharing 65% and 38% amino acid similarities, respectively (Table S5). M-52 and rat astrovirus shared only 43% and 19% similarities in ORF1b and ORF2, respectively. M-52 is therefore the first characterized mouse astrovirus species.

### Rodent adeno-associated virus and adenovirus

Adeno-associated viruses (AAV) are small, ssDNA viruses with icosahedral capsid symmetry belonging to the *Dependovirus* genus of the family *Parvoviridae*. A characteristic of AAVs is their dependence on co-infection with adenovirus as a helper virus for their replication. AAV genomes are ca 4.7-kb in length and contain two major ORFs, and 145-bp inverted terminal repeats, which serve as the origins of replication [94]. AAV contains two major ORFs encoding the nonstructural Rep proteins and structural Cap proteins. Adenoviruses (AdV) are non-enveloped viruses composed of an icosahedral capsid and a double-stranded linear DNA genome ranging from 26 to 45-kb in length.

In our study, we found both AAV and AdV sequences in one deer mouse (*Peromyscus maniculatus*) fecal sample (M-6). The AAV sequence reads resulted in three separate contigs (GenBank JF755424-JF755426), covering 40% (304 amino acids) of the AAV VP7 protein showing the highest similarity to porcine AAV (61%) and AAV-5 (60%), followed by rat AAV (55%). It shared a lower similarity with mouse AAV-1 of only 43%. The phylogenic tree confirmed that AAV M-6 clustered with porcine AAV, AAV-5 and rat AAV (Figure S2A). AdV sequence reads, consisted of two separate contigs encoding 10% (154 amino acids) of the AdV hexon protein. M-6 (GenBank JF755423) exhibited the closet match of 80% to murine AdV2 with which it clustered phylogenetically (Figure 2B).

### Insect viruses

Thirty-nine percent of the rodent virome sequences were related to insect viruses (Figure 1). Detection of these viral sequences may be due to insect consumption. Insect RNA virus matches were more abundant than insect DNA viruses, making up 67% of insect viral sequences. Of note, 72% of RNA sequences were related to chronic bee paralysis virus (CBPV, an unassigned member of the *Dicistroviridae* family or of a distinct family in the picornavirales) known to infect honeybees [95]. Feces of infected honeybees are positive for CBPV (91). Other sequences also belonged to the family *Dicistroviriade* and had closest matches to newly identified viruses, including kelp fly virus and Nora virus [96], [97]. Insect DNA viruses from the viral families *Iridoviridae* and *Polydnaviridae* and the subfamily *Densovirinae* were found. The majority of insect virus-like sequences shared protein similarity of less <70% with annotated insect viral proteins.

### Plant viruses

About 3.4% of the rodent virome (911 sequence reads) were related to plant viruses. DNA viruses were predominant with 86% of the reads and the largest proportion was single-stranded DNA viruses from the family *Nanoviridae* (48%), followed by the families *Geminiviridae* (12%) and *Phycodnaviridae* (7.2%). Interestingly, 33% of the DNA viral sequences were related to fungi infecting *Sclerotinia sclerotiorum* hypovirulence-associated DNA virus 1 (SsHADV-1), the first reported mycovirus with a DNA genome [98]. RNA viruses accounted for only 14% of plant viral sequences and the majority was related to single-stranded RNA viruses in the family *Secoviridae* (64%), followed by the families *Tymoviridae*, *Alphaflexiviridae* and *Tombusviridae*. Double-stranded RNA viruses were responsible for 28% of the plant viral RNA sequences, belonging to the family *Partitiviridae*.

## Discussion

Animal and human viral discovery has long been focused on pathogenic infections and viruses that could be readily grown in cell cultures and cause visible cytopathic effects. Viral metagenomics is a recent approach to analyzing mixtures of viral nucleic acids enriched directly from a variety of sources including animals, plants, protozoa, bacteria, archaea, and diverse environments without a prerequisite in vitro or in vivo amplification [20], [50]–[52], [99]–[110]. Viral metagenomics has been successfully employed to identify both commensal viruses and viral pathogens and has the potential to detect all viruses recognizable through sequence similarity searches [111]. We describe here the fecal viral flora in several species of wild rodents. Sequences closely and distantly related to known viral sequences were identified as well as many sequences of unknown taxonomic origin [51], [99], [101], [108], [112]–[114]. A fraction of these currently unclassifiable sequences may be derived from still genetically uncharacterized viral families refractory to nucleotide or protein sequence similarity based searches. Multiple known DNA and RNA viral families infecting plants, insects, and mammals were also detected. Plant and insect viral sequences likely reflect the omnivorous diet of these rodents.

The number of Rep-containing circular DNA genomes recently found in various animal and environmental viral surveys has greatly expanded the genetic diversity in this group of viruses [56], [62], [115]–[119]. Some of the Rep proteins of the small circular DNA genomes characterized here clustered with viruses in marine environment or in protozoan genomes. Type II RodSCVs contained two separate Rep ORFs in their genomes, separately encoding the viral Rep and RNA helicase with each gene individually smaller than those in single Rep containing genomes. The many novel circular DNA genomes seen here indicate the likely existence of several previously unknown viral families. The closest relatives of some Rep sequences found in rodent feces are those integrated in the *Giardia intestinalis* genome, possibly reflecting their replication in protozoans in rodent guts.

We report on the first identification of a kobuvirus in mice (*Peromyscus crinitus* and *P. maniculatus*). Since its genetic characterization in 1998 in Japan [65], Aichi virus, the archetypical kobuvirus, has been associated with acute gastroenteritis worldwide [74], [120], [121]. Other kobuvirus species have been reported in pigs and cows as well as in humans and associated with diarrhea [68]–[70], [121]. Our phylogenetic analysis revealed that the mouse kobuvirus clustered with human Aichi virus. A recent study describes a similar close relationship between a canine kobuvirus and Aichi virus [122]. This close relationship between human, mouse, and canine kobuviruses suggests past zoonotic transmission of kobuviruses between these hosts or their recent ancestors followed by independent evolution leading to the close but distinct species seen today.

*Sapelovirus* is another genus in the family *Picornaviridae* and currently includes 3 species (www.picornaviridae.com). While phylogenetically related to the enteroviruses, these viruses have a different type of internal ribosome entry site and a leader protein (www.picornaviridae.com). A Sapelovirus-like genome fragment was identified in house mouse feces. Phylogenetically, this fragment clustered with the other Sapeloviruses. Mice therefore also appear to harbor Sapeloviruses, expanding the known host range of this group of viruses.

Two potential new picornaviridae genera we provisionally labeled Mosavirus and Rosavirus were genetically characterized. Only distantly related to the kobuvirus genus, the Rosavirus is also part of a larger clade containing several avian picornaviruses, including the recently described avian turdiviruses and turkey hepatitis virus [93], [123]. The recent characterization of numerous deep-branched members of the picornaviridae family (likely new genera) from mammals, birds, reptiles, and fish indicates this viral family is still greatly under sampled and likely to rapidly expand as more potential host species are analyzed (www.picornaviridae.com). A second potential new genus distantly related to the cardioviruses was also characterized. Until recently cardioviruses consisted of the Theiloviruses and Encephalomyocarditis viruses infecting largely rodents and the Saffold virus group infecting humans [124]–[126]. The Mosavirus also exhibited the large genetic distance relative to other picornaviruses required to tentatively qualify Mosavirus as a founding member of a new Picornaviridae genus.

Using consensus PCR primers PBV has been detected in the feces of mammals, birds, and snakes with or without diarrhea [127]–[132]. Infection was common in wild rodents with 22/105 animals shedding PBV-like nucleic acids, the second highest group of mammalian virus-like sequences after the highly diverse small circular DNA genomes (Figure 1). PBV therefore represent the largest quantity of viral nucleic acids released by these rodents into the environment. Two nearly complete PBV RdRp sequences clustered together with a larger clade of human PBV cluster I sequences possibly reflecting past rodent-human transmission. Complete PBV RdRp sequences from more animal species will be required to further test this hypothesis.

The number of mammalian astroviruses species has recently undergone a rapid expansion [85], [88]–[91], [133], [134]. The first astrovirus genome from a mouse clustered with a clade containing mink, sheep, and recently identified human astroviruses [89], [91]. The detection of multiple unrelated astrovirus species within some host species (human, pigs, and sea lions) may reflect frequent cross-species transmission rather than diversification from a common source.

AAV infects humans and other primate species [16], [135], [136]. Although over 80% of humans are seropositive, AAV has not been associated with any diseases in humans [137]. Members of the family *Adenoviridae* infect various species of vertebrates, including humans [51], [138]–[140]. The majority of AdV infection cause upper respiratory diseases; however, AdV also causes other symptoms, such as acute gastroenteritis [141]. The small AAV and AdV fragments identified here are too limited for definitive classification but provisionally appear to represent a fourth species of murine adenovirus [142].

Rodents are known reservoirs of numerous viruses capable of causing human diseases [2]. The characterization of the viromes of animal species with frequent contacts with humans can provide baseline viral content to more quickly identify the possible sources of future zoonotic infections and therefore assist in their control. Changes in the baseline virome of various animal may also assist in identifying changes associated with future population crashes. The addition of annotated viral genomes to public databases will also facilitate their inclusion on microarrays as well as assist in their precise identification after high-throughput sequencing [143], [144]. Monitoring for rodent viruses in humans will require extensive geographical sampling in persons with high exposure such as hunters, hikers, and farmers, and in regions of high biodiversity. The large increase in rodent viral species and higher level taxa revealed here by a limited species, geographic, and individual sampling reflects the large extent of mammalian virus diversity awaiting characterization.

## Materials and Methods

### Rodents and samples

Fecal specimens were collected from 52 mice, 52 voles and one Woodrat and stored at −80°C. Rodents were humanely captured and released according to the international guidelines of the American Society of Mammalogists (www.mammalsociety.org). Four mouse species *Peromyscus crinitus* (Canyon mouse), *P. maniculatus* (Deer mouse), *P. truei* (Pinyon mouse), and *P. boylii* (Brush mouse) were trapped in three different California counties, Siskiyou, El Dorado and Lassen, in May and June 2010 (Table 1). Two species of voles were analyzed: one *Microtus longicaudus* (Long-tailed vole) was caught in El Dorado Co., CA in June 2010 and 51 *Microtus pennsylvanicus* (Meadow vole) plus 20 *Mus musculus* mice were caught in an old field habitat in southeastern Virginia during April-November 2008. A *Neotoma cinerea* (Woodrat) was trapped in Siskiyou Co., CA in May 2010. The feces collected from the traps were used in this study. Sample collection was exempted from review by the IACUC committees of the CDPH and of the Old Dominion University.

**Table 1:** Summary of rodent sample information.

Rodent	Species (No. of rodent)	Collection location	Collection time
Mouse	*Peromyscus crinitus* [Cryon mouse] (7)*; Peromyscus maniculatus* [Deer mouse] (20)*; Peromyscus truei* [Pinyon mouse] (1); *Peromyscus boylii* [Brush mouse] (4)	Siskiyou, El Dorado and Lassen, California	May-June, 2010
	*Mus musculus* [House mouse] (20)	Southeastern Virginia	April-November, 2008
Woodrat	*Neotoma cinerea* (1)	Siskiyou, California	May, 2010
Vole	*Microtus longicaudus* [Long-tailed vole] (1)	El Dorado, California	June, 2010
	*Microtus pennsylvanicus* [Meadow vole] (51)	Southeastern Virginia	April-November, 2008

### Viral particle purification and nucleic acid extraction

Fecal samples were resuspended in Hanks buffered saline solution (Gibco BRL) and vortexed. The samples were clarified by 15,000 x *g* centrifugation for 10 min. A total of 200 µl of supernatants was filtered through a 0.45-µm filter (Millipore) to remove bacterium-sized particles. The filtrate was then treated with a cocktail of DNases (Turbo DNase from Ambion, Baseline-ZERO from Epicentre, and Benzonase from Novagen) and with RNase (Fermentas) to digest unprotected nucleic acids [52]. Nucleic acids protected from nuclease digestion within viral capsids were then extracted using QIAamp spin-columns according to the manufacturer's instructions (Qiagen).

### Viral metagenome library construction for 454 pyrosequencing

cDNA synthesis was performed as described previously [52]. Briefly, RNA only and DNA plus RNA virus sequence-independent amplifications were separately performed and then combined before sequencing. For RNA virus-only amplification, 10 µl of the extracted nucleic acid was treated with DNase (Ambion) and was used as a template to synthesize cDNA, using SuperScript III reverse transcriptase (Invitrogen) and a primer containing an arbitrary set sequence followed by a randomized eight nucleotides at the primer's 3′ end. For the RNA plus DNA virus amplification, the DNase step prior to RT was excluded. Following reverse transcription followed by heat denaturation and re-annealing of the primer a single round of primer extension DNA synthesis was performed using Klenow fragment polymerase (New England Biolabs). PCR amplification was then performed using primers consisting of only the set portion of the random primer. To increase sampling of the viral nucleic acids, the random PCR amplifications were performed in duplicate resulting in four PCR products (2 from viral RNA-only and 2 from viral RNA plus DNA). The four PCR products were pooled and purified using the QIAquick Purification Kit (Qiagen). The purified DNA level was determined by Nanodrop (Thermo Scientific). Equal amounts of amplified DNA from up to 40 different fecal samples (using different primers subsequently recognizable by their set sequences) were combined into larger pools to generate 3 libraries. A total of 120 µg of DNA from each library was run on a 2% agarose gel, yielding a DNA smear. DNA ranging in apparent size from 500 to 1,000-bp was cut from the gel and purified using the QIAquick Gel Extraction Kit (Qiagen). The extremities of the PCR products DNA were then polished using T4 polynucleotide kinase. The Roche/454 adaptors were then ligated, and small DNA fragments removed, according to the manufacturer's protocol (GS FLX Titanium General Library Preparation Kit, Roche).

### Sequence read classification

The set sequences on the different random PCR primers were used to assign sequence reads to the corresponding fecal samples. Sequence reads were trimmed of their set primer sequences and adjacent eight nucleotides corresponding to the randomized part of the primers. Trimmed sequences from each bin were then de novo assembled into contigs using Sequencher (Gene Codes), with a criterion of at least 95% identity over 35-bp to merge two fragments. The assembled sequence contigs and singlet sequences greater than 100-bp were compared to the GenBank nonredundant nucleotide and protein databases using BLASTn and BLASTx, respectively. Based on BLAST output, sequences were classified as viruses, phage, bacteria, and eukaryota based on the taxonomic origin of the best-hit (lowest E score) sequence match. An E value of 10^−5^ was the cutoff value for significant hits. Sequences whose best alignment E value was >10^−5^ were deemed unclassifiable.

### Complete genome sequencing of circular DNA viruses

Complete circular DNA viral genomes were amplified using inverse PCR (iPCR) with specific primers designed from 454 derived short-sequence fragments. iPCR amplicons were then directly sequenced by primer walking. PCR reactions contained 2.5 U of LA Taq polymerase (Takara) in 2.5 µl of 10X LA PCR buffer (Takara), 4 µl of 2.5 mM dNTP (Takara), 2.5 µl of forward and reverse primers (10 pmol/µl), and 2.5 µl of nucleic acids (for first round) or 1 µl of the first-round PCR product (for second PCR round) as a template in a 25 µl total volume. PCR was performed at 94°C for 1 min, followed by 30 cycles of 98°C for 10 s, 68°C for 4–10 min depending on the sizes of the expected amplicon, and a final extension at 72°C for 10 in, and then held at 4°C.

### Genome acquisition and detection of linear RNA viruses

All primers are described in Table S6. Sequence reads showing significant BLASTn or BLASTx hits to Aichi virus were linked together using RT-PCR. The 5′ and 3′ rapid amplification of cDNA end (5′ and 3′ RACE) was used to acquire the 5′ and 3′ extremities of the Aichi-like virus genome [89], [145]–[147]. For the complete genome of astrovirus, pairs of specific reverse (Ast-R1 and Ast-R2) and forward primers (Ast-F1 and Ast-F2) designed from an initial astrovirus-like-sequence of 414-bp were used in 5′ and 3′ RACE [89], [145]–[147] to amplify ∼1.5-kb and 5-kb PCR products, respectively. In a mouse (*Peromyscus crinitus*) fecal specimen two small picornavirus-like-genome fragments (300-bp and 251-bp, respectively) were detected. PCR failed to link these two fragments together, suggesting that they belonged to two different viruses. For the complete genome of the Mosavirus, specific reverse (Mosa-R1 and Mosa-R2) and forward primers (Mosa-F1 and Mosa-F2) were used in 5′ and 3′ RACE [89], [145]-[147] to amplify ∼1.5-kb and 6-kb PCR products, respectively. For the Rosavirus, a pair of specific forward primers (Rosa-F1 and Rosa-F2) were used in 3′ RACE [89], [146], [147] to amplify ∼4-kb amplicon. 5′ RACE was not successful. To investigate the prevalence of Aichi-like virus, consensus primers were used for PCR screening designed on a nucleotide alignment of the 2C-3B region of all human Aichi virus genotypes available in GenBank and the mouse Aichi-like virus strain characterized here. For the RT reaction, 10 µl of extracted RNA was added to 10 µl of RT mixture containing 4 µl of 5X First-Strand buffer (Invitrogen), 1 µl of 10 mM dNTP (Fermentas), 1 µl of random primer, 1 µl of SuperScript III Reverse Transriptase (Invitrogen), 1 µl of RNase inhibitor (Fermentas), and 1 µl of DEPC-treated water. The RT reaction mixture was incubated at 25°C for 5 min, 50°C for 60 min, 70°C for 15 min to inactivate the enzyme, and then held at 4°C. For the first round PCR, 2.5 µl of cDNA template was mixed with 2.5 µl of 10X ThermoPol Reaction buffer (New England Biolabs), 0.5 µl of 10 mM dNTP (Fermentas), 2.5 µl of each primer (10 pmol/µl) (Ai-Deg-F1 and Ai-Deg-R1), targeting the Achi-like virus, 0.4 µl of Taq DNA Polymerase (New England Biolabs). DEPC-treated water was added up to a 25 µl total volume. The PCR condition was as follows: denaturation at 95°C for 5 min, 35 cycles of 95°C for 30 s, 63°C for 30 s and 72°C for 1 min, a final extension at 72°C for 10 min, and then held at 4°C. The second round of amplification was performed using the same conditions except that the annealing temperature was 60°C, and inner primers Ai-(Deg-F2 and Ai-Deg-R2). The second round PCR amplification resulted in the amplicon size of 735-bp.

### Phylogenetic analysis and genomic structure prediction

Reference viral sequences from different viral families were obtained from GenBank. Sequence analysis was performed using CLUSTAL X with the default settings. Sequences were trimmed to match the genomic regions of the viral sequences obtained in the study. A phylogenetic tree with 100 bootstrap resamples of the alignment data sets was generated using the neighbor-joining method [148]. The genetic distance was calculated using Kimura's two-parameter method (PHYLIP) [149]. Sequence identity matrix was measured using BioEdit. GenBank accession numbers of the viral sequences used in the phylogenetic analyses were shown in the trees. Putative ORFs in the genome were predicted by NCBI ORF finder. The circular genome architectures were predicted using Vector NTI 11.5 Advance (Invitrogen) with the following conditions: minimum ORF size of 100 codons, start codons ATG and GTG, stop codons TAA, TGA and TAG. To identify stem-loop structures, nucleotide sequences were analyzed with Mfold. Full and partial genome sequences are at GenBank accession numbers JF755401-JF755427, and JF973686-JF973687. The 454 pyrosequencing data is in the short read archive at SRA030869.

## Supporting Information

Figure S1 **Phylogenetic analysis of mouse Sapelovirus.** Phylogenetic tree obtained from partial P1 protein of the genera Enterovirus, Sapelovirus, and Hepatovirus in the family *Picornaviridae*. The novel Sapelovirus is labeled with a black circle.
(PDF)

Figure S2 **Phylogenetic analysis of mouse adeno-associated virus (AAV) and adenovirus. A.** Phylogenetic tree obtained from partial VP7 protein of AAVs. The novel AAV is labeled with a black circle. **B**. Phylogenetic tree obtained from hexon protein of adenoviruses. The novel adenovirus is labeled with a black circle.
(PDF)

Table S1 **PmPV1 nucleotide (amino acid) sequence similarity (%) to other papillomaviruses belonging to the different genera.**
(PDF)

Table S2 **Genomic characteristics of novel circular DNA viruses detected in rodents.**
(PDF)

Table S3 **Coding potential/putative proteins of the genome of mouse kobuvirus and comparison of amino acid sequence similarity (%) of the eleven proteins of a newly discovered mouse kobuvirus, human Aichi virus and bovine kobuvirus that belong to the** ***Kobuvirus*** **genus in the family of** ***Picornaviridae*** **.**
(PDF)

Table S4 **Pairwise amino acid sequence similarity (%) between P3 regions of Mosavirus, Rosavirus and their closely-related picornavirus genera.**
(PDF)

Table S5 **Pairwise amino acid sequence similarity (%) between novel mouse astrovirus, its closely related astroviruses, and rat astrovirus.** The upper right and lower left were ORF2 (capsid) and ORF1b (RdRp) similarities, respectively.
(PDF)

Table S6 **Primers used in the study.**
(PDF)
