# Supplementary figures and images for: The Fecal Viral Flora of Wild Rodents
Source: PLoS Pathog. 2011 Sep 1;7(9):e1002218. doi: 10.1371/journal.ppat.1002218 (PMC3164639; doi:10.1371/journal.ppat.1002218)

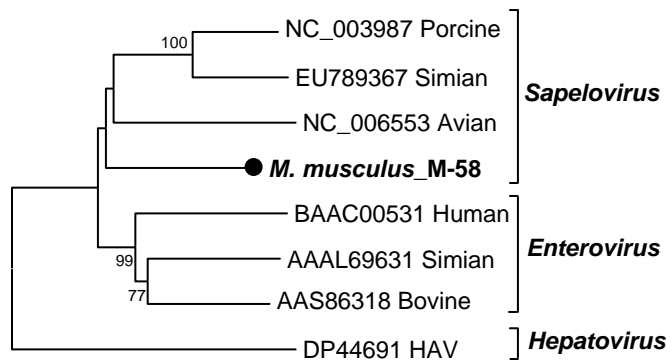

0.1

Supplement: Figure S1 — Phylogenetic analysis of mouse Sapelovirus. Phylogenetic tree obtained from partial P1 protein of the genera Enterovirus, Sapelovirus, and Hepatovirus in the family Picornaviridae. The novel Sapelovirus is labeled with a black circle. (PDF) [file ppat.1002218.s001.pdf]

**A**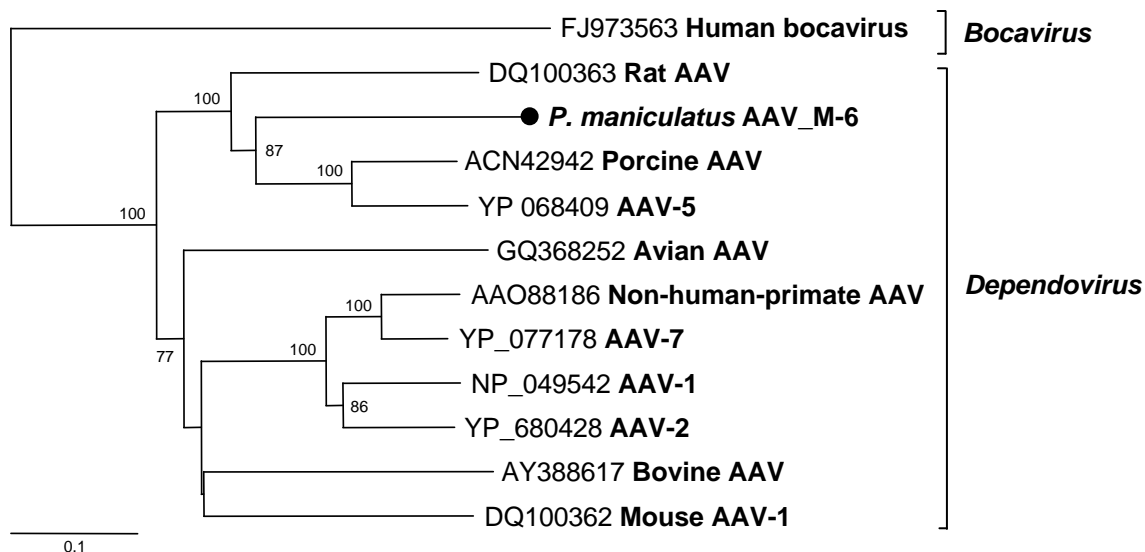**B**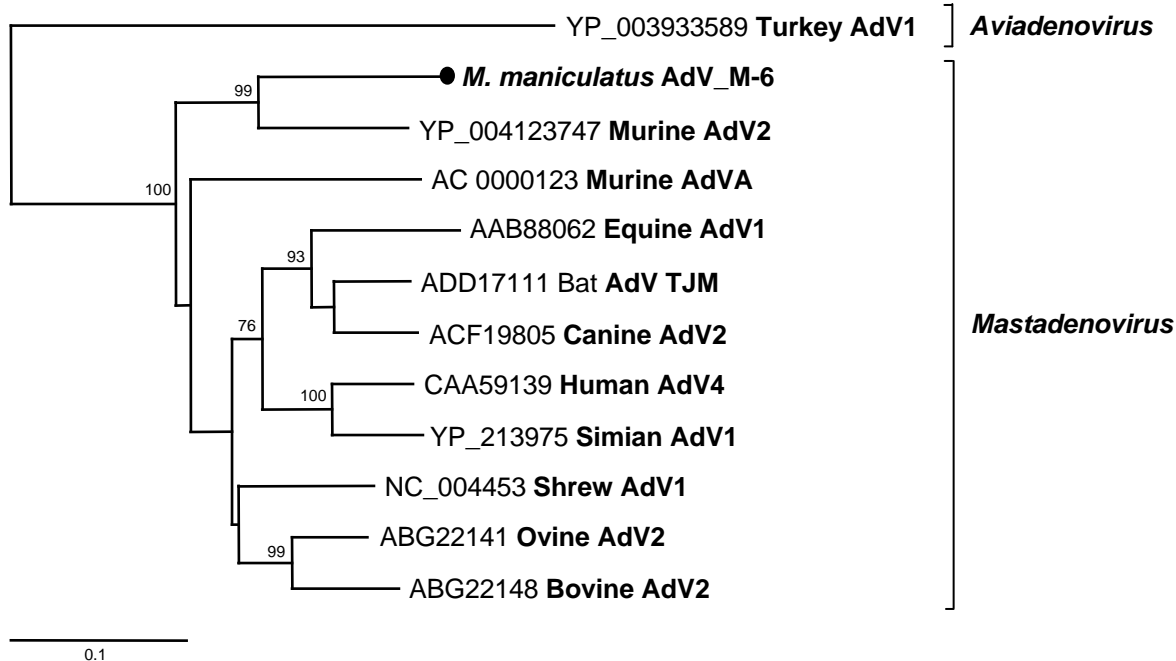

Supplement: Figure S2 — Phylogenetic analysis of mouse adeno-associated virus (AAV) and adenovirus. A. Phylogenetic tree obtained from partial VP7 protein of AAVs. The novel AAV is labeled with a black circle. B. Phylogenetic tree obtained from hexon protein of adenoviruses. The novel adenovirus is labeled with a black circle. (PDF) [file ppat.1002218.s002.pdf]
