# Supplementary material for: The Fecal Viral Flora of Wild Rodents
Source: PLoS Pathog. 2011 Sep 1;7(9):e1002218. doi: 10.1371/journal.ppat.1002218 (PMC3164639; doi:10.1371/journal.ppat.1002218)
Supplement: Table S1 — PmPV1 nucleotide (amino acid) sequence similarity (%) to other papillomaviruses belonging to the different genera. (PDF) [file ppat.1002218.s003.pdf]

| ORF PmPV1 | MnPV1 | EePV1 | TmPV1 | MmiPV1 | HPV41 | EdPV1 | CPV1 | BPV5 |
|---|---|---|---|---|---|---|---|---|
| **E6** | 52 (45) | 39 (34) | 40 (30) | 34 (23) | 31 (22) | 30 (18) | 40 (31) | 30 (19) |
| **E7** | 45 (35) | 37 (32) | 33 (29) | 46 (30) | 35 (25) | 27 (13) | 48 (33) | 25 (12) |
| **E1** | 64 (61) | 48 (43) | 49 (39) | 53 (43) | 42 (30) | 43 (32) | 47 (40) | 46 (37) |
| **E2** | 43 (42) | 32 (30) | 32 (26) | 27 (26) | 29 (19) | 30 (21) | 35 (22) | 29 (21) |
| **L2** | 51 (46) | 37 (27) | 33 (22) | 42 (26) | 33 (21) | 31 (20) | 33 (23) | 31 (21) |
| **L1** | 63 (67) | 56 (55) | 54 (51) | 57 (54) | 47 (39) | 53 (50) | 50 (51) | 53 (21) |
