# Supplementary material for: The Fecal Viral Flora of Wild Rodents
Source: PLoS Pathog. 2011 Sep 1;7(9):e1002218. doi: 10.1371/journal.ppat.1002218 (PMC3164639; doi:10.1371/journal.ppat.1002218)
Supplement: Table S2 — Genomic characteristics of novel circular DNA viruses detected in rodents. (PDF) [file ppat.1002218.s004.pdf]

Circular virus	Rodent species	Accession number	Nonamer sequence	Stem length (bp)	No. of nt in genome	No. of aa in Rep protein	Family domains in Rep protein	Top hit (E-value)	Similarity (%)
M-13	*P. truei*	JF755410	GGGTAATAC	14	2,193	270	Viral Rep; RNA helicase	Giardia intestinalis (4×10^{-35})	29
R-15	*N. cinerea*	JF755401	TAATATTAC	17	1,758	345	Gemini-AL1; RNA helicase	Porcine circovirus 2 (7×10^{-14})	20
M-44	*M. musculus*	JF755408	AAGTAATAC	18	2,294	309	Viral Rep; RNA helicase	Reclaimed water circovirus-like genome RW-E (1×10^{-73})	44
M-45	*M. musculus*	JF755409	GGGTAATAC	11	2,506	203	Viral Rep	Reclaimed water circovirus-like genome RW-A (2×10^{-19})	20
M-53	*M. musculus*	JF755415	GCGTCTTAC	8	1,124	Rep 1: 149	Viral Rep	Marine circovirus-like genome CB-A (1×10^{-20})	20
						Rep 2: 125	RNA helicase	Giardia intestinalis (2×10^{-18})	12
V-64	*M. pennsylvanicus*	JF755407	CTCAGTTAC	18	2,986	Rep 1: 174	No conserved domain	Geminiviruses (3×10^{-2})	10
						Rep 2: 192	RNA helicase	Bat circovirus-like genome TM-6c (6×10^{-14})	12
V-69	*M. pennsylvanicus*	JF755403	GACCCTTAC	10	2,220	321	Viral Rep; RNA helicase	Giardia intestinalis (2×10^{-89})	44
V-72	*M. pennsylvanicus*	JF755411	CTTTACTAC	17	2,070	313	Viral Rep; RNA helicase	Giardia intestinalis (2×10^{-82})	52
V-76	*M. pennsylvanicus*	JF755404	TGCCGTTAC	11	3,781	349	Gemini-AL1; RNA helicase	Giardia intestinalis (6×10^{-16})	20
V-77	*M. pennsylvanicus*	JF755405	TGCCGTTAC	11	3,781	349	Gemini-AL1; RNA helicase	Giardia intestinalis (6×10^{-16})	20
V-81	*M. pennsylvanicus*	JF755412	CTTTACTAC	17	2,070	313	Viral Rep; RNA helicase	Giardia intestinalis (2×10^{-82})	52
V-84	*M. pennsylvanicus*	JF755413	CTTTACTAC	17	2,070	313	Viral Rep; RNA helicase	Giardia intestinalis (2×10^{-82})	52
V-86	*M. pennsylvanicus*	JF755416	CTCAGTTAC	18	2,984	Rep 1: 193	No conserved domain	Geminiviruses (3×10^{-2})	10
						Rep 2: 192	RNA helicase	Bat circovirus-like genome TM-6c (6×10^{-14})	12
V-87	*M. pennsylvanicus*	JF755406	TGCCGTTAC	11	3,781	349	Gemini-AL1; RNA helicase	Giardia intestinalis (6×10^{-16})	20
V-89	*M. pennsylvanicus*	JF755402	GGGTAATAC	8	2,069	309	Viral Rep; RNA helicase	Giardia intestinalis (5×10^{-79})	50
V-91	*M. pennsylvanicus*	JF755417	CTCAGTTAC	18	2,984	Rep 1: 193	No conserved domain	Geminiviruses (3×10^{-2})	10
						Rep 2: 192	RNA helicase	Bat circovirus-like genome TM-6c (6×10^{-14})	12
V-97	*M. pennsylvanicus*	JF755414	CTTTACTAC	17	2,070	313	Viral Rep; RNA helicase	Giardia intestinalis (2×10^{-82})	52
