# Supplementary material for: The Fecal Viral Flora of Wild Rodents
Source: PLoS Pathog. 2011 Sep 1;7(9):e1002218. doi: 10.1371/journal.ppat.1002218 (PMC3164639; doi:10.1371/journal.ppat.1002218)
Supplement: Table S3 — Coding potential/putative proteins of the genome of mouse kobuvirus and comparison of amino acid sequence similarity (%) of the eleven proteins of a newly discovered mouse kobuvirus, human Aichi virus and bovine kobuvirus that belong to the Kobuvirus genus in the family of Picornaviridae . (PDF) [file ppat.1002218.s005.pdf]

Mouse kobuvirus			Human Aichi virus	Bovine kobuvirus
**Putative protein**	**Position**	**Length (aa)**		
**L**	M^{1} - Q^{172}	172	68	35
**P1**			**81**	**53**
**VP0**	G^{173} - P^{547}	375	84	60
**VP3**	Q^{548} - Q^{771}	224	90	62
**VP1**	S^{772} - Y^{1048}	277	70	38
**P2**			**84**	**61**
**2A**	V^{1049} - Q^{1159}	111	71	64
**2B**	G^{1160} - Q^{1324}	165	81	54
**2C**	G^{1325} - Q^{1659}	335	89	69
**P3**			**84**	**63**
**3A**	G^{1660} - Q^{1754}	95	68	42
**3B**	A^{1755} - Q^{1781}	27	78	54
**3C**	G^{1782} - Q^{1971}	190	81	49
**3D**	S^{1972} - A^{2439}	468	89	76
