# Supplementary material for: The Fecal Viral Flora of Wild Rodents
Source: PLoS Pathog. 2011 Sep 1;7(9):e1002218. doi: 10.1371/journal.ppat.1002218 (PMC3164639; doi:10.1371/journal.ppat.1002218)
Supplement: Table S4 — Pairwise amino acid sequence similarity (%) between P3 regions of Mosavirus, Rosavirus and their closely-related picornavirus genera. (PDF) [file ppat.1002218.s006.pdf]

**1. Mosavirus**	ID												
**2. Teschovirus**	**25**	ID											
**3. Erbovirus**	**25**	26	ID										
**4. Senecavirus**	**27**	31	27	ID									
**5. Cardiovirus**	**26**	28	27	44	ID								
**6. Aphthovirus**	**28**	23	23	30	26	ID							
**7. Cosavirus**	**26**	30	28	35	35	27	ID						
	**1**	**2**	**3**	**4**	**5**	**6**	**7**	**8**	**9**	**10**	**11**	**12**	**13**
**8. Rosavirus**								ID					
**9. Kobuvirus**								**30**	ID				
**10. Turdivirus 1**								**29**	45	ID			
**11. Turdivirus 2**								**31**	40	39	ID		
**12. Salivirus**								**28**	43	37	33	ID	
**13. THV**								**28**	27	27	29	26	ID
