# Supplementary material for: The Fecal Viral Flora of Wild Rodents
Source: PLoS Pathog. 2011 Sep 1;7(9):e1002218. doi: 10.1371/journal.ppat.1002218 (PMC3164639; doi:10.1371/journal.ppat.1002218)
Supplement: Table S5 — Pairwise amino acid sequence similarity (%) between novel mouse astrovirus, its closely related astroviruses, and rat astrovirus. The upper right and lower left were ORF2 (capsid) and ORF1b (RdRp) similarities, respectively. (PDF) [file ppat.1002218.s007.pdf]

Species	1	2	3	4	5	6	7
**1. Mouse AstV**	ID	38	30	26	28	27	17
**2. Bat AstV**	65	ID	30	26	28	27	18
**3. Ovine AstV**	47	50	ID	37	40	40	17
**4. Mink AstV**	46	51	60	ID	39	38	17
**5. HMO AstV-A**	47	52	62	61	ID	48	16
**6. Human AstV-AV1**	48	53	62	61	73	ID	19
**7. Rat AstV**	43	48	47	48	48	48	ID
