# Supplementary material for: The Fecal Viral Flora of Wild Rodents
Source: PLoS Pathog. 2011 Sep 1;7(9):e1002218. doi: 10.1371/journal.ppat.1002218 (PMC3164639; doi:10.1371/journal.ppat.1002218)
Supplement: Table S6 — Primers used in the study. (PDF) [file ppat.1002218.s008.pdf]

Name	Orientation	Sequence (5’-3’)
AstF1	Forward	TGACGATCAAATCACATACGCAGT
AstF2	Forward	GGATTTGCTGCCGGGTGTGT
AstR1	Reverse	CCCTATATTGTTGTTCTGTCCA
AstR2	Reverse	ACACACCCGGCAGCAAATCC
Mosa-F1	Forward	GATTGCCCACAGACATGA
Mosa-F2	Forward	TTTGATCCTGCCATACCTG
Mosa-R1	Reverse	GAGCACCATGAACACCAAG
Mosa-R2	Reverse	CTCATGTCTGTGGGCAATC
Rosa-F1	Forward	AATATGCTCCAGCGCACCTA
Rosa-F2	Forward	ACTGCACGGTGATTCGGAAGG
Ai-Deg-F1	Forward	CGTCCACCTKCSGATCAACGTCACCTC
Ai-Deg-F2	Forward	GGAGGGTGGCCCARTCGTAGG
Ai-Deg-R1	Reverse	CTCAACCCMATCCCYGGACCCAA
Ai-Deg-R2	Reverse	AGCGGGARAAGAKRTAGAAGCCAGAC

Note: K is G or T; S is C or G; R is A or G; M is A or C; Y is C or T.
